# Palmitic Acid Esterification Boosts Epigallocatechin Gallate’s Immunomodulatory Effects in Intestinal Inflammation

**DOI:** 10.3390/biom15081208

**Published:** 2025-08-21

**Authors:** Raúl Domínguez-Perles, Concepción Medrano-Padial, Cristina García-Viguera, Sonia Medina

**Affiliations:** Laboratorio de Fitoquímica y Alimentos Saludables (LabFAS), CSIC, CEBAS, Campus Universitario de Espinardo, Edificio 25, 30100 Murcia, Spain; rdperles@cebas.csic.es (R.D.-P.); conmedpad@gmail.com (C.M.-P.); smescudero@cebas.csic.es (S.M.)

**Keywords:** lipophenols, intestinal epithelium, macrophage, immunomodulation, interleukins, cellular phenotype, inflammation

## Abstract

Lipophenols, combining phenolic and lipid moieties in a single molecule, are valuable candidates for providing enhanced bioactive properties with therapeutic potential, including anti-inflammatory functions associated with immune-mediated diseases such as intestinal bowel disease (IBD). Thus, palmitoyl–epigallocatechin gallate (PEGCG), a lipophilic derivative of epigallocatechin gallate (EGCG), has been highlighted for its enhanced stability in lipid-rich environments and bioavailability due to improved cellular uptake. However, the contribution of lipophilic esterification to PEGCG’s capacity to inhibit inflammation and the development of harmful autoimmune responses remains underexplored. This work uncovered the differential efficiency of EGCG and its palmitoyl derivative in modulating, in vitro, the interleukin profile generated by intestinal epithelium under inflammatory conditions. Therefore, both could attenuate the immune response by lowering macrophage migration and polarisation towards pro-inflammatory (M1) or anti-inflammatory (M2) phenotypes. While the fatty acid moiety gave PEGCG a functional advantage over EGCG in adjusting the interleukin-based response of intestinal epithelium to inflammation—since both of them decreased, to a similar extent, the expression of pro-inflammatory interleukins, namely IL-6, IL-17, IL-18, IL-23, and TNF-α (which lowered by 11.2%, on average)—the former was significantly more efficient in cushioning the increase in IL-1β and IL-12p70 (by 9.2% and 10.4%, respectively). This immune modulation capacity did not significantly impact the migration and expression of costimulatory molecules featuring M1 (CD86^+^) or M2 (CD206^+^) phenotypes by THP-1-derived macrophages, for which both bioactive compounds exhibited equivalent efficiency. Nonetheless, the analysis of the pro- and anti-inflammatory interleukins secreted by differentiated macrophages allowed the identification of an advantage for PEGCG, which decreased the expression of the pro-inflammatory immune mediators IL-1β and IL-12p70, IL-23, and TNF-α more efficiently. These results suggest that lipophilisation of phenolic compounds presents exciting potential for extending their application as functional molecules by combining the effects of their polar head with their ability to interfere with membranes, conveyed by their lipophilic tail. In addition, the enhanced reactivity would confer a higher capacity to interact with cellular signalling molecules and thus inhibit or attenuate the immune response, which is of special interest for preventing the onset and severity of immune-mediated pathologies such as IBD.

## 1. Introduction

To date, the prevalence of inflammatory bowel disease (IBD) has increased worldwide, reaching up to 6.8 million in 2017, boosted by the Westernised lifestyle and dietary habits [1], with forecasts predicting that 1% of the population will be living with IBD by 2030 [2]. IBD involves a group of chronic inflammatory conditions of the gastrointestinal tract, mainly Crohn’s disease and ulcerative colitis, which are characterised by periods of remission and flare-ups. This pathology, which severely disrupts quality of life [3], has been associated with malfunctioning interactions between the immune system, intestinal microbiota, and a range of stressors [4,5]. Among the most investigated environmental factors in IBD are dietary components, which play a crucial role in modulating the microbiota and, consequently, contribute to triggering autoreactive immune responses. In this frame, specific combinations of factors activate the migration and differentiation of immune cells that infiltrate local tissues [6], causing auto-reactive damage [7]. However, diets can also provide molecules that decrease the severity of these processes. Among the dietary factors influencing the course of IBD, phytochemical compounds potentially interact with immune cells’ receptors, giving rise to attenuated symptoms [8,9,10,11].

The assessment of (poly)phenols on the biological scope, as paradigmatic phytochemicals responsible for the anti-inflammatory attributes of plant-based foods, has been celebrated specifically for epigallocatechin gallate (EGCG) concerning a range of biological properties (e.g., anti-inflammatory) [12]. EGCG is a natural catechin abundantly present in green tea, with recognised antioxidant and anti-inflammatory activities. Given its biological value, esterification alternatives have drawn significant attention as they contribute to overcoming the limitations associated with the phenolics’ hydrophilicity, including reduced bioavailability, limited membrane permeability, and a restricted functional scope under physiological conditions [13]. However, the biological advantages of lipid esterifications, particularly in the form of palmitoyl–epigallocatechin gallate (PEGCG), remain underexplored. Lipophenols or phenolipids are a distinctive class of phenolic compounds known for the lipophilic character conferred by the fatty acid moiety that allows combining the chemical advantages of lipids and phenolics in a single compound with remarkable physicochemical and biological properties [14]. This makes them highly versatile in biological systems, conferring an enhanced capacity to cross the cell membranes and react with molecular targets [15,16,17,18], thus acting efficiently as anti-inflammatory molecules [19].

**Figure 1:**
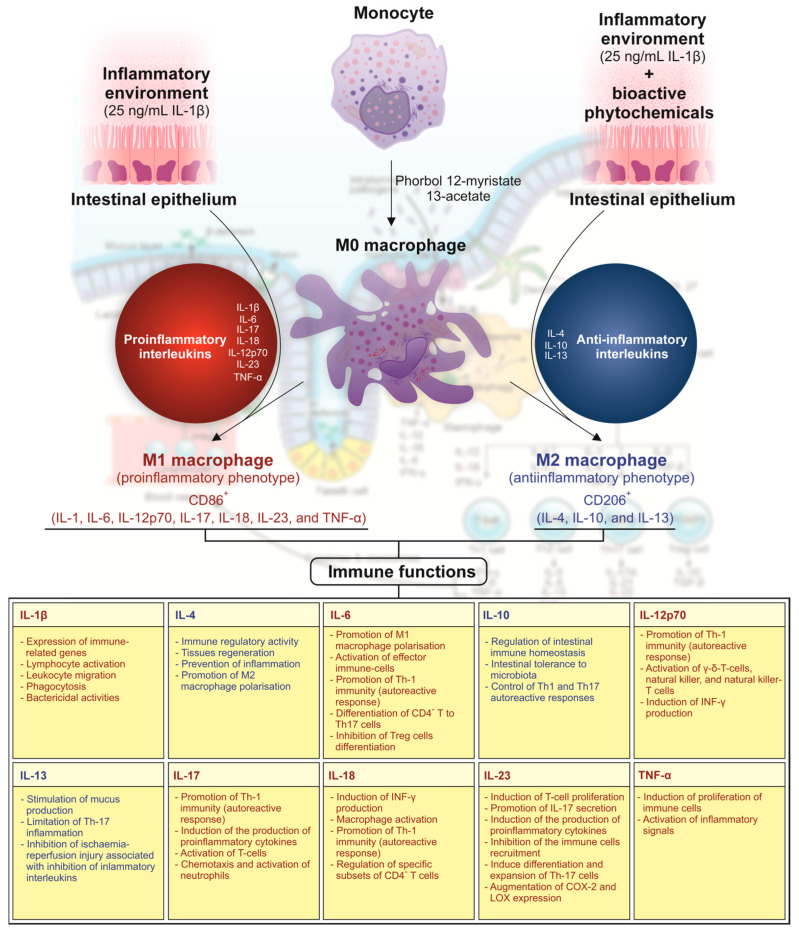
Interleukins involved in the differentiation and function of myelomonocytic cells and the orchestration of autoreactive immune response, featuring intestinal bowel disease.

The literature available on EGCG esterified with fatty acid (e.g., docosapentaenoic acid) has reported greater anti-inflammatory activity than native catechin due to a more efficient inhibition of human cyclooxygenase-2 (COX-2) and the subsequent production of oxylipins involved in the inflammatory process [20]. Nonetheless, although it has been hypothesised that this activity is mirrored in the interleukin environment, which governs the activation of the adaptive immune response upon the differentiation of macrophages towards antigen-presenting cells (APCs), this fact remains underexplored [4]. Hence, IBD is associated with the secretion of augmented levels of pro-inflammatory cytokines (e.g., IL-1β, IL-6, and TNF-α), which drive cellular infiltration and tissue damage [21]. Interleukins secreted by the intestinal epithelium in inflammatory conditions play a central role in immune regulation, thus controlling the course of IBD. Given the relevance of intercellular signalling for building an auto-reactive immune response, an enhanced understanding of the regulatory functions and counterbalance of subsets of APCs that interact through interleukins with the damaged tissue and effector cells offers opportunities for adjusting immune interventions [22]. This is especially relevant for modulating the scope of the immune response, particularly targeting these signalling molecules or their receptors. In this context, the effect of the presence/absence of modulating compounds (e.g., EGCG and PEGCG) on macrophage migration to the inflammatory focus through fine-tuning the quantitative profile of interleukins involved in chemotactic events deserves assessment (Figure 1).

In this environment, and depending on the specific interleukin profile, macrophages can differentiate into two distinct phenotypes, M1-CD86^+^ or pro-inflammatory (activated by stimuli like lipopolysaccharide (LPS) and interferon-γ (IFN-γ)) and M2-CD206^+^ or anti-inflammatory (induced by IL-4 and IL-13) macrophages, which develop activating or attenuating functions on the immune response, respectively [6,23]. This polarisation defines the overall progression of IBD [21,24]. Therefore, identifying differentiation factors would help to attenuate its symptoms, as the M1 and M2 balance is crucial for resolving the inflammatory status [24].

Recent studies have suggested that EGCG may modulate macrophage polarisation by reducing the M1 macrophage subtype and promoting the frequency of the M2 phenotype, thus demonstrating varied cellular and molecular effects, which depend on the microenvironment [25,26]. However, esterified forms of EGCG (e.g., PEGCG) may offer new and enhanced avenues for therapeutic interventions, yet their effects on macrophage behaviour in inflammatory conditions require further investigation. To date, no studies have evaluated the role of PEGCG on macrophage polarisation or cytokine modulation in intestinal inflammation, representing a critical gap in the current literature.

According to these antecedents, the present study investigates the enhanced capacity of PEGCG to decrease the migration and differentiation of intestine resident macrophages relative to the native EGCG, focusing on the capacity to fine-tune the interleukin microenvironment and the induction of specific anti-inflammatory phenotype (M2), responsible for mitigating the autoreactive role of the adaptive immune response featuring the IBD pathogenesis.

## 2. Materials and Methods

### 2.1. Chemicals and Reagents

Trypsin-ethylenediaminetetraacetic acid (EDTA), Eagle’s Minimum Essential Medium (EMEM), L-glutamine, foetal bovine serum (FBS), penicillin/streptomycin, and essential amino acids were sourced from Gibco (ThermoFisher Scientific, Madrid, Spain). Flat-bottom 24-well plates and polycarbonate membrane inserts (5 µm pore size) were obtained from Corning (New York, NY, USA). (−)-Epigallocatechin gallate (EGCG) (No. CAS 989-51-5) and lipopolysaccharide (LPS) were obtained from Sigma–Aldrich (St. Louis, MO, USA). Phorbol 12-myristate 13-acetate (PMA), IL-1β, IL-4, and IL-13 were sourced from R&D Systems (Minneapolis, MN, USA). CoraLite^®^ Plus 647 Anti-Human CD86 and CD206 antibodies were obtained from Proteintech^®^ (ThermoFisher Scientific, Madrid, Spain; Catalog #CL647-65165 and #CL647-65155, respectively). All LC-MS solvents were obtained from J.T. Baker (Phillipsburg, NJ, USA). Ultrapure water was produced using a Millipore^TM^ water purification system (Bedford, MA, USA).

Invitrogen ELISA kits were utilised for the quantification of various human interleukins: IL-4 (Catalog #KHC0041), IL-6 (Catalog #EH2IL6), IL-10 (Catalog #EHIL10), TNF-α (Catalog #KHC3011), IL-12p70 (Catalog #KAC1568), IL-13 (Catalog #BMS231-3), IL-23 (Catalog #EH268RB), IL-17 (Catalog #EH260RB), and IL-1 (Catalog #EH256RB) were purchased from Invitrogen-Thermofisher Scientific (Madrid, Spain). Additionally, Proteintech provided the human IL-13 ELISA kit (Catalogue #KE00020).

### 2.2. Cell Lines, Culture, and Experimental Conditions

The human colon adenocarcinoma (Caco-2, HTB-37^TM^) and human monocytic (THP-1, TIB-202^TM^) cell lines were sourced from the American Type Culture Collection (ATCC^®^, Rockville, MD, USA) and maintained at passage numbers between 20 and 30 for the experiments.

Caco-2 cells were grown following the described methodology to obtain a monolayer model of the intestinal barrier [10] that constitutes an in vitro system mimicking the absorptive and barrier functions of human intestinal epithelium. Upon completion of the cellular monolayer, cells were detached using Trypsin-EDTA at 0.05%. The culture medium used was EMEM supplemented with 10% heat-inactivated FBS, 1% non-essential amino acids, 1% penicillin/streptomycin (5000 U/mL), and 2 mM L-glutamine.

THP-1 cells were grown using the culture medium RPMI 1640 supplemented with 10% FBS, penicillin/streptomycin (5000 U/mL), and 2 mM L-glutamine (final concentration). To ensure compatibility with the experimental conditions between the two cell lines, THP-1 cells were adapted to the growth medium of Caco-2 cells (EMEM medium).

### 2.3. Macrophage Migration Assay

To sort out the relative capacity of PEGCG to modulate macrophage migration in comparison with EGCG, the former was synthesised and characterised according to the published procedures [27,28]. Briefly, the chemical lipophilisation of EGCG with palmitoyl chloride to obtain the ester derivative PEGCG was performed by adding EGCG (100 mg) to 2.20 mL of dry acetone. Sodium acetate anhydrous and a 1.05 molar ratio of palmitoyl chloride were added dropwise under an N_2_ atmosphere and mechanical stirring in darkness and at room temperature (RT). The reaction mixture was stirred for 5 h and monitored by TLC to confirm the formation of PEGCG and consumption of EGCG. Afterwards, the resulting mixture was filtered through Celite^®^ 545 (Sigma-Aldrich, St. Louis, MO, USA), washed with 2.00 mL of acetone, and concentrated under reduced pressure to a faint yellow oily product. The acylation reaction product (150 mg) was dissolved in 19 mL of an acetonitrile/LC-MS water mixture (80:20, *v*/*v*) and purified by preparative HPLC/UV-*vis* (Model Agilent Infinity II equipped with Agilent 1290 Infinity II fraction collector, Agilent Technologies, Waldbronn, Germany). The column used for the chromatographic resolution was an Agilent Prep-C18 Scalar (4.6 × 250 mm, 10 µm, Agilent Technologies, Waldbronn, Germany), at RT, using mobile phases A and B, LC-MS water and acetonitrile, respectively. The flow rate and injection volume were 1 mL/min and 400 µL, respectively. The target lipophenolic was resolved chromatographically upon the following elution gradient (time, %B): (0 min, 5%), (6 min, 10%), (12 min, 15%), (18 min, 20%), (24 min, 50%), (30 min, 80%), and (40 min, 90%), returning to the initial conditions at 50 min. The PEGCG was monitored using UV detection at 275 nm, which provides the maximum absorption of EGCG and ester derivatives, and the collection was initiated automatically. All fractions collected throughout 5 independent runs were pooled. Then, the solvent was removed through rotary evaporation at 25 °C and analysed using UHPLC-ESI-QqQ-MS/MS, UHPLC-ESI-Q-ToF-MSn, and nuclear magnetic resonance (^1^H and ^13^C NMR) to evaluate the efficiency of the purification process and obtain comprehensive identification of PEGCG.

The products obtained from the esterification reaction were characterised using an HPLC 1200 series model (Agilent Technologies, Waldbronn, Germany) equipped with a diode array detector (DAD) and a Bruker HCT Ultra ion trap mass detector (Bruker Daltonics, Bremen, Germany) in series. A Luna C18 column (250.0 × 4.6 mm, 5.0 μm particle size; Phenomenex, Torrance, CA, USA) was used, and the mobile phases and gradients were described above. The flow rate and injection volume were 800 µL/min and 20 µL, respectively. Spectral data were accumulated in the 240–330 nm range, and the chromatograms were analysed at 275 nm. Nitrogen was used as nebulising gas at a pressure of 65.0 psi, and the flow was adjusted to 11 L/min. The ionisation conditions were fine-tuned at 350 °C and 4.5 kV for capillary temperature and voltage, respectively. The full-scan mass covered the range from *m*/*z* 100 to *m*/*z* 1200. Collision-induced fragmentation experiments were performed using helium as a collision gas in the ion trap, with voltage ramping cycles from 0.3 to 2.0 eV. The MS data were acquired in negative ionisation mode, and the MS(*n*) was carried out, automatically, on the most abundant fragment ion in MS(*n* − 1). The acylation reaction products were monitored, and the synthesised PEGCG was identified by comparing the mass fragments, elution order, and UV–*vis* spectra obtained with those provided by the authentic standard of EGCG and described in the literature.

The newly synthesised compound and the EGCG authentic standard were dissolved in DMSO (stock solution) to further supplement the cells’ growth media (described in Section 2.2) at a 95.5:0.5 (*v*/*v*) rate, and a solvent control (0.5% DMSO) was included in the experimental design, according to the conditions applied in a previous characterisation of the cytotoxicity of EGCG and PEGCG [29].

Caco-2 cells were treated with 1 µM EGCG or PEGCG (0.458 and 0.697 μg/mL, respectively) in growth media for 1 h, followed by treating cells with 25 ng/mL IL-1β for 10 h, when the highest level of immune modulatory interleukins is secreted [30]. Doses were normalised on a molar basis to ensure equal numbers of molecules were applied across treatments, thus allowing direct comparison of bioactivity between EGCG and PEGCG. Although this results in slightly different mass-based concentrations, the molar approach was considered more appropriate for evaluating structure–activity relationships [31]. Untreated Caco-2 cells were used as negative controls, and Caco-2 cells exposed only to 25 ng/mL IL-1β were assigned as positive controls. This study applied 1 μM EGCG and PEGCG to identify potential immunomodulatory functionality. Hence, the evaluation of such concentrations provided results that offer a more accurate view of their biological impact and therapeutic relevance in vivo, as stated by Singh et al. [32]. Thus, while the highest concentrations assessed can induce cellular responses, they may also cause unintended cytotoxic effects [33], leading to opposite interpretations of their therapeutic potential.

The migration of THP-1-derived macrophages in response to chemotactic signals produced by Caco-2 cells under inflammatory conditions was assessed using a 24-well polycarbonate membrane insert with a 5 µm pore size by applying the methodology described in the literature [34]. Briefly, in the basolateral chamber of the Transwell^®^ plate (Corning, New York, NY, USA), 600 µL supernatant of Caco-2 cells was collected after treating cells with 1 µM EGCG or PEGCG in Caco-2 growth media. Subsequently, 300 µL of THP-1 cells at a concentration of 5 × 10^5^ cells/mL were added to the apical chamber (Transwell^®^ insert) and incubated for 24 h at 37 °C with 5% CO_2_. Afterwards, the Transwell^®^ inserts were carefully removed from the wells. The medium containing the migrated cells in the bottom chamber was gently mixed, and cells were counted using blue trypan staining in a Neubauer counting chamber. Migrating cells were expressed as a percentage of the positive control.

### 2.4. Assessment of the Capacity of Palmitoyl Epigallocatechin Gallate to Modulate THP-1 Monocytic Cell Differentiation Towards M1 and M2 Phenotypes

THP-1 monocytes were seeded into 12-well plates at a concentration of 10^6^ cells/mL and treated with PMA at 150 nM for 24 h to differentiate monocytes into M0 macrophages. This was conducted to identify the capacity of the interleukin microenvironment generated by Caco-2 cell growth under proinflammatory conditions (25 ng/mL IL-1β), in the presence/absence of EGCG or PEGCG, to induce polarisation of THP-1 derived M0 macrophages, which were exposed to Caco-2 cells’ supernatant obtained as a result of the diversity of treatments detailed in Section 2.3 for 24 and 72 h to assess the interleukins environment on the capacity to induce M1 and M2 polarisation, respectively.

The M1 and M2 phenotypes were determined by two-colour flow cytometry. With this objective, cells were harvested, washed with PBS, and centrifuged at 500× *g* for 5 min. The supernatant was discarded, and the pellets obtained were incubated for 30 min with mouse anti-human Coralite^®^ Plus-647 fluorochrome-labelled antibodies CD86 (for M1 pro-inflammatory macrophages) and CD206 (for M2 anti-inflammatory macrophages) at the concentrations recommended by the manufacturer (5 µL per 10^6^ cells in 100 µL of PBS). After incubation, the cells were washed up and centrifuged at 500× *g* for 5 min. The supernatants were discarded, and cells were resuspended in PBS with 0.1% sodium azide and 0.5% bovine serum albumin (BSA). Flow cytometry was performed with a FACScalibur cytometer (Becton Dickinson, San Diego, CA, USA), and data were analysed with FlowJo v10.10 software (Becton Dickinson, San Diego, CA, USA). A total of 5000 events per sample were collected, and the Mean Fluorescence Intensity (MFI) was determined for each marker and experimental condition.

### 2.5. Profiling Interleukin Environments

The secretion of the pro-inflammatory (IL-1β, IL-6, IL-12p70, IL-17, IL-18, IL-23, and TNF-α) and anti-inflammatory (IL-4, IL-10, and IL-13) interleukins was assessed in the supernatant obtained with the experimental conditions detailed in Section 2.3 and Section 2.4 using enzyme-linked immunosorbent assay (ELISA) kits following the procedure recommended by the manufacturer to ensure the reliability of cytokine measurements.

### 2.6. Statistical Analysis

All experimental conditions were performed in triplicate (*n* = 3), and the data were expressed as the mean ± standard deviation (SD). According to the normal distribution and homogeneity of variance of the data (determined by the Shapiro–Wilk (<50 samples) and Levene tests, respectively), the obtained results were subjected to one-way analyses of variance (ANOVA). When statistical differences were identified, the variables were compared using Duncan’s multiple range test. Significant differences were set at *p* < 0.05. All statistical analyses were performed with SPSS, version 29.0 (SPSS Inc., Chicago, IL, USA).

## 3. Results and Discussion

This study highlights the enhanced capacity of a lipophilic catechin derivative (PEGCG) as a modulator of the immune cascade underlying intestinal inflammation compared to the native EGCG. This assessment builds upon prior work that achieved the chemical synthesis and characterisation of PEGCG [27], and demonstrated its improved anti-inflammatory activity relative to EGCG, upon the modulation of the expression of COX-2 and related isoprostanoids, a key pathway in the initiation and propagation of intestinal inflammation. However, given the immunological component of inflammatory diseases such as IBD, sorting out the differential ability of both compounds to modulate the recruitment of immune-competent cells into the inflammasome and polarise the sign of the immune response (pro-inflammatory vs. anti-inflammatory) would allow retrieving a more comprehensive picture of the tentative advantages conferred by the fatty acid moiety, in terms of prevention of the inflammatory process due to the tentatively acquired amphiphilic character (despite its aqueous solubility remaining underexplored) by the esterification of EGCG with palmitic acid. In this context, enhanced lipophilicity alone can represent a major constraint for the organic distribution of bioactive molecules. Although the aqueous solubility of PEGCG has not been fully established, previous studies on amphiphilic EGCG monoesters, such as 4″-C14 EGCG and 4″-C16 EGCG, suggest that this class of derivatives can preserve both biological activity and sufficient dispersion in aqueous environments to exert their effects, thereby supporting the potential of PEGCG as a functionally relevant compound despite its increased lipid affinity [35]. With this purpose, the present work comprehensively addressed the interleukin profile secreted by intestine epithelium under inflammatory conditions, the capacity of EGCG and PEGCG to modulate the macrophage migration into the inflammatory focus, and the polarisation towards pro-inflammatory (M1) and/or anti-inflammatory (M2) phenotypes by determining the expression of specific clusters of differentiation and interleukin profile responsible for modulating the course of inflammation.

### 3.1. Interleukin Profile Produced by Intestinal Epithelium After Treating Cells with EGCG and PEGCG

As referred to before, IBD is associated with persistent mucosal inflammation that is determined by uncontrolled immune responses [36]. The global incidence and prevalence of IBD have risen over the past decade worldwide, highlighting a significant public health challenge [37], which has boosted the need for developing novel treatments. To explore the capacity of bioactive phytochemicals, such as catechins, and prevent IBD medical conditions, the application of the inflammatory stimulus (IL-1β) allowed the reproduction of changes in intestinal homeostasis, and the activation of the inflammatory response and tissue damage featuring IBD [36], through the recruitment of immune cells to the gut mucosa, and their differentiation towards activated immune cells [38]. These events result from the secretion of a range of interleukins (among other signalling molecules) by the intestinal epithelium, with an effect on adhesion molecules and macrophage migration [39,40]. Thus, to evaluate EGCG and PEGCG as anti-inflammatory and immunomodulatory molecules, selecting molar bases of them (1 μM equivalent to 0.458 and 0.697 μg/mL, respectively) as the primary reference allowed us to directly compare EGCG and PEGCG at the molecular level, which is particularly relevant when assessing functional modifications and structure–activity relationships [31].

#### 3.1.1. Modulation of Pro-Inflammatory Interleukins Secreted by a Caco-2-Based Intestinal Barrier Under Inflammatory Conditions

**Figure 2:**
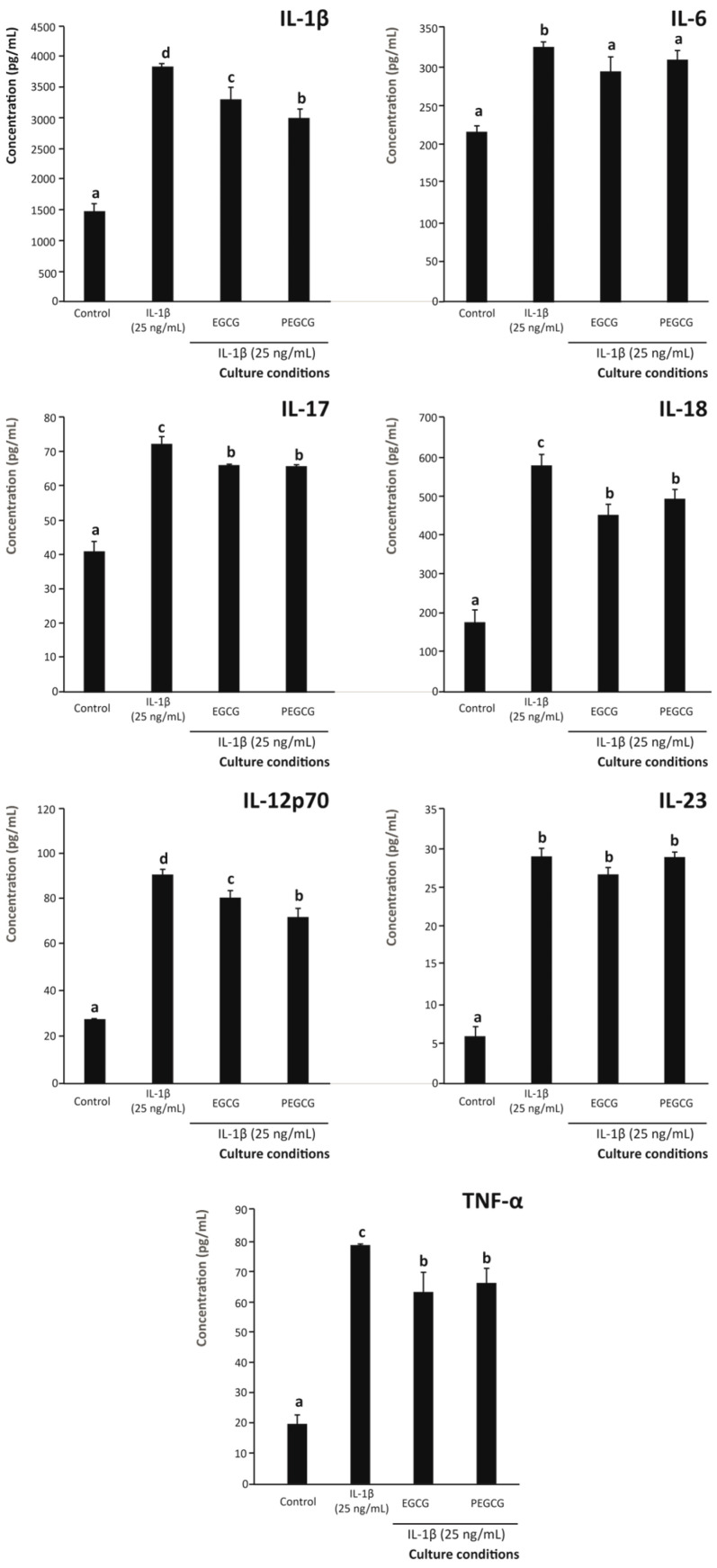
Capacity of the 1 µM EGCG and PEGCG to modulate the secretion of pro-inflammatory interleukins (IL)-1β, IL-6, IL-17, IL-18, IL-12p70, IL-23, and TNF-α by Caco-2 cells exposed to an inflammatory stimulus (25 ng/mL IL-1β). Distinct lowercase letters within each bar plot indicate significantly different values at *p* < 0.05 according to one-way analyses of variance (ANOVA) and Duncan’s multiple range test (*n* = 3).

When assessing the pro-inflammatory interleukins secreted by intestinal epithelium, quantifiable amounts of IL-1β, IL-6, IL-12p70, IL-17, IL-18, IL-23, and TNF-α were recorded. For all of them, both EGCG and PEGCG reduced the secretion by the epithelial cells by up to 21.9% compared with cells exposed to the inflammatory stimulus without the intervention of any anti-inflammatory compound (Figure 2). Among the positive control (25 ng/mL of IL-1β) and the same stimulus with EGCG and PEGCG, the decline observed was significant (*p* < 0.05) for all assessed interleukins, except for IL-23.

In addition, when comparing the specific efficiency of ECGC and PEGCG to lower the secretion of interleukins, the effect observed was not significantly different (*p* > 0.05) for IL-6 (7.3% lower, on average), IL-17 (8.8% lower, on average), IL-18 (18.3% lower, on average), IL-23 (4.1% lower, on average), and TNF-α (17.6% lower, on average) (Figure 2).

According to these results, in general, both EGCG and its palmitoyl derivative allowed, at least, slowing down the development of the immune component of inflammation by reducing the secretion of pro-inflammatory interleukins, in good agreement with accumulating evidence engaging the interleukin profile and the suppression of excessive inflammatory response in the intestinal mucosa as a key pathogenic component of IBD [41]. Specifically, the decreased secretion of IL-6, which mediates the inflammatory response upon binding to gp130 of target cells, thus triggering the subsequent signalling cascade responsible for recruiting macrophages to the inflammasome, lowers the production of additional inflammation-related chemokines and inhibits the invading ability of myeloid cells [42]. In addition, the modulatory effect of IL-6 on the immune response is also associated with the production of immunoglobulin A (IgA) by differentiated B cells [43] that actively contribute to the initiation of inflammation at mucosal sites, namely the intestinal wall [44]. This inflammatory mediator contributes to defining the interleukin profile by boosting the secretion of TNF-α and IL-1β, which are pivotal elements controlling the immunological cascade associated with chronic inflammatory disorders (e.g., IBD) [44]. Therefore, the inhibition of IL-6 secretion by both EGCG and PEGCG would contribute to tackling the inflammatory cascade by a range of mechanisms.

IL-17 is characterised by immunological properties related to mucosal protection against pathogens [45] that, when dysregulated, can give rise to autoimmune diseases [46]. In this regard, the significant reduction in the IL-17 levels by both EGCG and PEGCG (Figure 2) would play a central role in preventing agents against the immunological cascade responsible for auto-reactive damage featuring Crohn’s disease [47]. However, the effects of IL-17 on intestinal epithelium are complex and to date, the lack of evidence on whether IL-17 is an effector or protector interleukin in the human intestinal epithelium needs to be clarified. Therefore, it remains essential to shed light on the IL-17-dependent mechanism responsible for the pathological effects on the intestinal epithelium and the possible mechanisms supporting its protective role against intestinal inflammation.

Another interleukin, promisingly modulated by EGCG and PEGCG, was IL-18 (belonging to the IL-1 family of interleukins [36]), which is implicated in the prevention of immune system malfunction associated with the pathogenesis of IBD, upon the ability to induce the secretion of IFN-γ [48]. Therefore, evidence on the capacity of EGCG and PEGCG to reduce the concentration of IL-18 informs on their potential to prevent intestinal inflammation. However, as referred to IL-17, the evidence available nowadays associates IL-18 with pleiotropic roles in the intestinal mucosa [49]. Therefore, the ability of both catechins to lower IL-17 and IL-18 further supports their potential to rebalance mucosal immunity. In this regard, additional research is needed to determine whether EGCG and PEGCG selectively modulate their secretion by epithelial and immune cells. Evidence in this regard will help to understand their actual relevance in preventing IBD.

To control the local immune response in the intestine and avoid autoreactive reactions against healthy and functional tissues, IL-23 contributes to maintaining a balance between tolerance and immunity in the intestine, thus being considered a key element in intestinal immune homeostasis [50]. Thus, the production of IL-23 within the intestine balances T-cell-dependent and -independent pathways, driving the immune response towards the production of Th1- and Th17-associated cytokines (related to autoimmune processes), which has suggested this immune mediator as a valuable therapeutic target, whose reduction could help to reduce the severity of IBD [51]. In the present study, neither EGCG nor its palmitoyl derivative significantly modulated its secretion, which contrasts with findings reported in other cellular models, where EGCG was associated with an increase in IL-23 levels through the modulation of alternative transcriptional pathways [52]. Such discrepancies may reflect differences in experimental conditions, including cell type, dose, or the complexity of the immune interactions. Specifically, models involving macrophages or systemic administration may trigger signalling mechanisms, such as STAT3-BATF2 or c-JUN/ATF2 activation, not fully recapitulated in epithelial cells [52].

The pathological mechanism of intestinal inflammation also involves the upregulation of TNF-α, which, in turn, augments the frequency of effector cells responsible for autoreactive events. Therefore, reducing the secretion of this cytokine would be an effective therapy for attenuating inflammation [41], since it would affect the frequency of T-reg cells in a tolerogenic way [53]. On the other hand, this cytokine contributes to mucin homeostasis and, thus, to protecting the intestinal tract against pathogens responsible for triggering the autoreactive immune response [54]. The moderate suppression of TNF-α by the target bioactive compounds assessed in the present work (Figure 2) suggests that they might act as adjuvant modulators rather than primary inhibitors.

In addition to the range of interleukins modulated in an IBD with a preventive purpose, with matching efficiency by EGCG and PEGCG, the latter exhibited a significantly (*p* < 0.05) enhanced the anti-inflammatory capacity in comparison with EGCG concerning the modulation of the secretion of two key inflammatory interleukins, namely IL-1β and IL-12p70 (by 9.2% and 10.4%, respectively) (Figure 2). The relevance of this finding is associated with the transversal functionality of IL-1β in inflammation, which has been described as a crucial mediator of autoimmune and inflammatory disorders [55]. Indeed, this interleukin controls the progression of the inflammatory status and the immune response characterising IBD by driving Th17 cell differentiation [56], which is closely related to the pathogenesis of IBD [36]. Finally, the differential secretion of IL-12p70 by intestinal epithelium under inflammatory conditions, when pre-treated with EGCG or PEGCG, constitutes a very relevant finding since this signalling molecule, commonly designated IL-12, constitutes a critical immunoregulatory interleukin that regulates adaptive immune responses by controlling the secretion of IFN-γ and forcing the differentiation of CD4^+^ T cells into Th1 cells [57]. Moreover, IL-12 shares biological functions with other family members (e.g., IL-23). Therefore, its efficient regulation will help to prevent IBD severity, and its specific modulation by PEGCG indicates a higher prevention capacity of the palmitoyl derivative compared with EGCG.

In summary, targeting IL-1β and IL-12p70 efficiently in an anti-inflammatory form by PEGCG allows the identification of a promising therapeutic approach against intestinal inflammation. Therefore, these results provide evidence supporting further research into IL-1β-associated post-transcriptional modifications, which would contribute to the elucidation of its intricate role in immunomodulation. Future work should dissect their precise molecular targets, such as IκB kinase, STAT3 phosphorylation, or NLRP3 inflammasome assembly, and evaluate combinatorial regimens in more complex co-culture or organoid models to validate translational potential.

#### 3.1.2. Modulation of Anti-Inflammatory Interleukins Secreted by a Monolayer Caco-2-Based Intestinal Barrier Under Inflammatory Conditions

**Figure 3:**
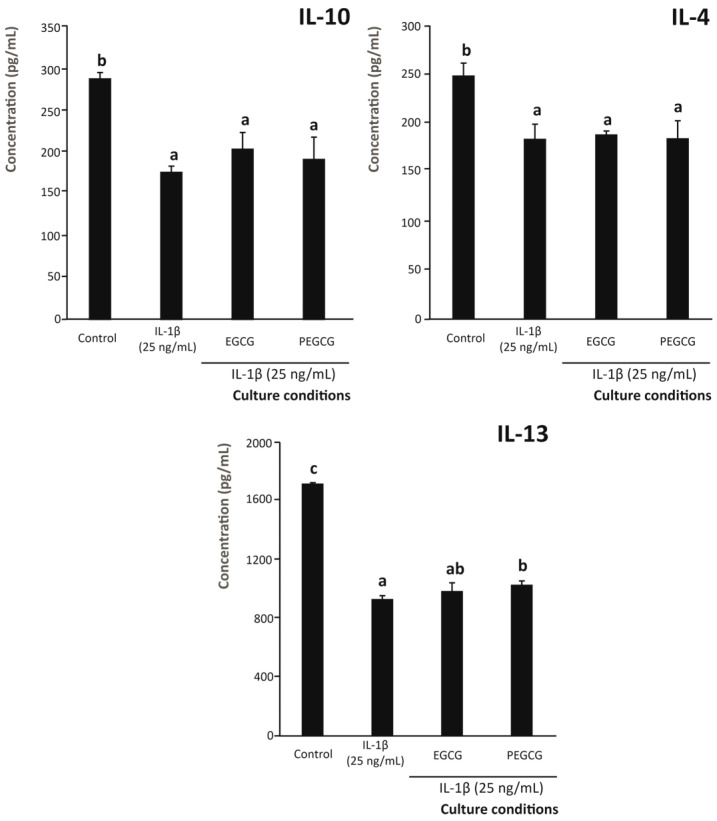
Capacity of the 1 µM EGCG and PEGCG to modulate the secretion of anti-inflammatory interleukins IL-10, IL-4, and IL-13 by Caco-2 cells exposed to an inflammatory stimulus (25 ng/mL IL-1β). Distinct lowercase letters within each bar plot indicate significantly different values at *p* < 0.05 according to one-way analyses of variance (ANOVA) and Duncan’s multiple range test (*n* = 3).

In addition to the pro-inflammatory interleukins, the effect on the level of anti-inflammatory signalisation by the intestinal epithelium was also assessed. In this regard, inflammatory conditions led to a significant decrease in the secretion of the anti-inflammatory interleukins IL-4, IL-10, and IL-13 compared to the control (unstimulated condition) (*p* < 0.05) (Figure 3). Similarly, pretreating Caco-2 cells with EGCG or PEGCG did not significantly restore IL-10 and IL-4 levels relative to untreated cells.

Regarding IL-13, pretreatment of the intestinal epithelium with PEGCG increased its concentration by 9.4% compared with the untreated control (although no significant difference was observed between PEGCG and EGCG) (Figure 3). This suggests that, under the tested conditions, the difference in the ability of PEGCG and EGCG to modulate the level of the tolerogenic IL-13 was negligible, thereby ruling out this pathway as an explanation for the enhanced anti-inflammatory and immunomodulatory capacity of PEGCG relative to EGCG.

These results contrast with previous evidence reported on the development of preclinical models that allowed describing that EGCG enhanced IL-10 and IL-4 levels under proinflammatory conditions, alleviating LPS-induced intestinal inflammation by inhibiting the TLR4/MyD88/NF-κB signalling pathway [58]. These discrepancies may be due to variations in the experimental conditions, the different biological settings of cell culture systems compared with in vivo models, and differences in dose and exposure time. On the other hand, the slight but significant effect on IL-13 levels caused by PEGCG suggested the development of improved bioactivity in the inflammatory environment compared to EGCG. In this sense, IL-13 affects epithelial tight junctions and barrier function [59]. These results advise that the esterification of EGCG with palmitic acid can enhance cell permeability or receptor interactions, enabling a targeted effect on specific interleukin expression. However, new dose–response and transcriptomic studies are required to reach definitive conclusions.

Given the crucial specific fine-tuning of the pro-inflammatory interleukin profile by PEGCG, next, the effect of the modified environment on the chemotaxis and differentiation of macrophages was evaluated to understand the actual capacity of the assessed lipophenols to tackle the molecular mechanisms involved in the pathogenesis of IBD.

### 3.2. Differential Capacity of PEGCG and EGCG to Modulate Macrophage Migration into the Inflammasome

As stated before, interleukins play a central role in immune regulation, thus controlling the course of IBD. Hence, an enhanced understanding of the regulatory functions and counterbalance of subsets of APCs that interact through interleukins with the damaged tissue and effector cells offers opportunities for adjusting immune interventions [22]. This is especially relevant for modulating the scope of the immune response, particularly targeting these signalling molecules or their receptors (Figure 1). In this context, the present work evaluated the effect of the presence/absence of modulating compounds (EGCG and PEGCG) on macrophage migration to the inflammatory focus by fine-tuning the quantitative profile of interleukins involved in chemotactic events.

**Figure 4:**
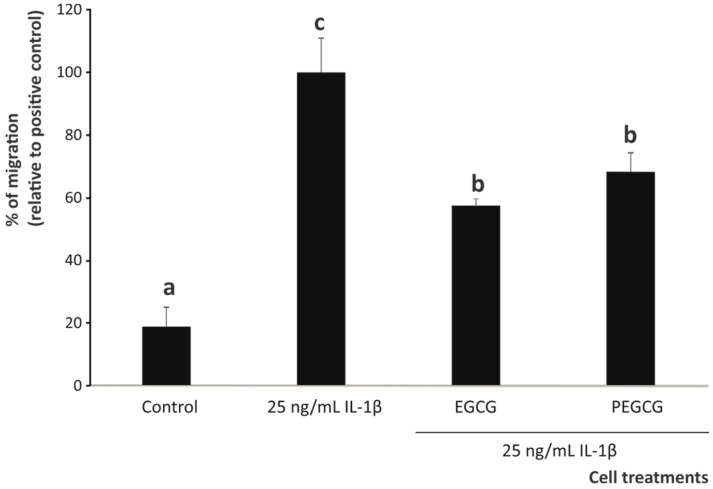
Capacity of epigallocatechin gallate (EGCG) and its esterified palmitoyl derivative (PEGCG) to modulate the percentage of migration of lamina propria resident macrophages induced by the interleukin cocktail produced by Caco-2 cells exposed to 25 ng/mL of IL-1β. Distinct lowercase letters indicate values significantly different at *p* < 0.05 according to one-way analyses of variance (ANOVA) and Duncan’s multiple range test (*n* = 3).

To achieve this objective, conditioned growth media from intestinal epithelium (Caco-2 cells) pretreated with EGCG and PEGCG, and exposed to an inflammatory environment, were assessed for the capacity to induce the migration of THP-1 cells (Figure 4). When monocytic cells were exposed to the inflammatory stimulus in the absence of preventive agents (positive control), the percentage of migration increased by 81.1% relative to unstimulated cells (control). Interestingly, pre-treating cells with EGCG and its palmitoyl derivative allowed for cushioning the referred migration rate, maintaining the level recorded for untreated cells despite the inflammatory environment. The comparison of both treatments evidenced no significant difference between the inhibition caused by EGCG and PEGCG (Figure 4).

The interaction of IL-6 with gp130 allows cells to trigger the subsequent signalling cascade responsible for recruiting macrophages into the inflammasome [60]. Thus, the inhibition of its secretion by EGCG, which fits well with previous descriptions in the literature of the immune regulatory power of this catechin derivative [61], is now extended to its palmitoyl derivative, which suggests the valuable contribution of this (poly)phenolic derivative.

Thus, both molecules lowered the production of inflammation-related chemokines and inhibited the invading ability of myeloid cells [42]. According to the current knowledge on the role of interleukins in macrophage migration, the capacity to inhibit the secretion of IL-6 would contribute to controlling the initiation of the autoreactive immune response triggered in the intestinal epithelium, according to the decrease in APC infiltration by modulating the production of immunoglobulin A [43] that has been enclosed in the inflammation initiation [44]. This inflammatory mediator contributes to defining the interleukin profile by boosting the secretion of TNF-α and IL-1β, which are pivotal elements controlling the immunological cascade and inflammatory disorders (e.g., IBD) [44].

In addition to the contribution of reduced IL-6 secretion by intestinal epithelium under inflammatory conditions, the macrophage migration inhibitory factor (MIF) is a chemotactic molecule ubiquitously expressed by various cells [62] that helps them recruit immune cells to settle inflammatory processes [63]. In this regard, to date, the relationship between IL-1β and MIF expression has been described as positively correlated [62]. Thus, the significant inhibition of the former by both EGCG and, to a greater extent, PEGCG could be responsible for the lowered macrophage chemotaxis recorded in the present work (Figure 4). This result is in agreement with previous descriptions of the EGCG capacity to suppress MIF expression in keratinocytes by Noh and Park (2012) [64], which reduces the recruitment of macrophages, responsible for initiating autoreactive immune responses. However, as far as we know, the present work describes, for the first time, the inhibition of macrophage migratory capacity by palmitoylated EGCG, although no advantage due to the inclusion of the fatty acid moiety in the chemical structure of EGCG was observed in line with the capacity to modulate the secretion of most pro-inflammatory interleukins, and specifically IL-6 (Figure 2 and Figure 4).

Moreover, IL-17 has been described as a chemoattractant for macrophages in specific locations [65] by inhibiting the migration of this cell type upon neutralising this interleukin using specific monoclonal antibodies [66]. Thereby, the significant reduction in IL-17 expression by epithelial cells treated with EGCG and PEGCG would help to reduce macrophage migration under inflammatory conditions [67]. This is associated with the expression of IL-18, which has been linked to the immune modulatory capacity of EGCG [64].

Finally, the contribution of TNF-α to the migration of macrophages to the inflammasome is mediated by its capacity to induce the generation of pro-inflammatory soluble factors that stimulate the mobilisation of THP-1 monocytes [68]. Thus, the similar capacity of EGCG and PEGCG to lower the secretion of this interleukin by epithelial cells (Figure 2) would give rise to the inhibition of macrophage recruitment (Figure 4) upon resolving the proinflammatory environment generated by epithelial cells exposed to IL-1β.

### 3.3. Polarisation of Macrophage Differentiation Towards M1/M2 Phenotypes by PEGCG vs. EGCG

Macrophages recruited into the inflammasome play a significant role in the pathophysiology of inflammation, which is dependent on their pro- or anti-inflammatory phenotype (M1 and M2, respectively). Therefore, after evaluating the capacity of EGCG and its palmitic acid derivative to modulate macrophage attraction, these phytochemicals’ capacity to polarise the differentiation of M0 macrophages into M1 or M2 cells was assessed by monitoring changes in the expression of specific clusters of differentiation (CD86 and CD206, respectively); this approach provides insights into macrophage behaviour and immune capacity, which is a cornerstone of deciphering the pathogenesis and potential treatments or preventive interventions for inflammatory disease.

#### 3.3.1. Contribution of PEGCG and EGCG to the Macrophage Polarisation Towards Pro-Inflammatory (M1) Cells

**Figure 5:**
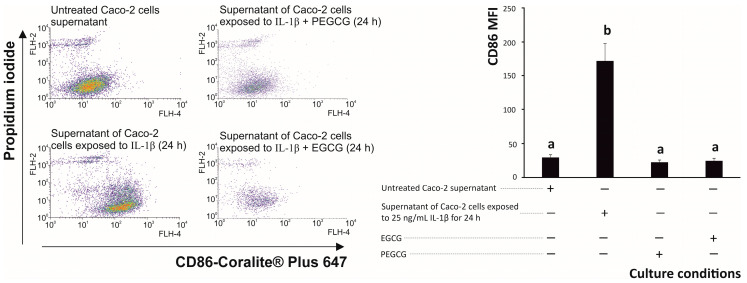
Dot plots of representative pro-inflammatory (CD86-Coralite^®^ Plus 647, M1) and propidium iodide double stains of M0 macrophages (differentiated from THP-1 monocytic cells treated with Phorbol-Myristate Acetate) exposed to pro-inflammatory growth media of Caco-2 cells in the presence/absence of epigallocatechin gallate and palmitoyl epigallocatechin gallate. Distinct lowercase letters indicate significantly different values at *p* < 0.05 according to one-way analyses of variance (ANOVA) and Duncan’s multiple range test (*n* = 3).

To investigate the relative efficiency of ECGC and PEGCG on the polarisation of THP-1-derived macrophage towards pro-inflammatory (M1) cells, after 24 h of incubation, the flow-cytometry-based analysis of the cell surface expression of CD86 was developed after exposition of THP-1 cells to the interleukins secreted by intestine epithelial cells, under inflammatory conditions, in the presence of EGCG and its palmitoyl derivative (Figure 5). Concerning the inflammatory settings (positive control), the interleukin microenvironment generated by epithelial cells after IL-1β stimulation significantly augmented the M1 marker CD86 [69].

Interestingly, the pre-treatment of Caco-2 cells with EGCG and PEGCG prevented the augmentation of the lowered CD86 expression that was maintained at the level expressed by untreated macrophages (Figure 5).

**Figure 6:**
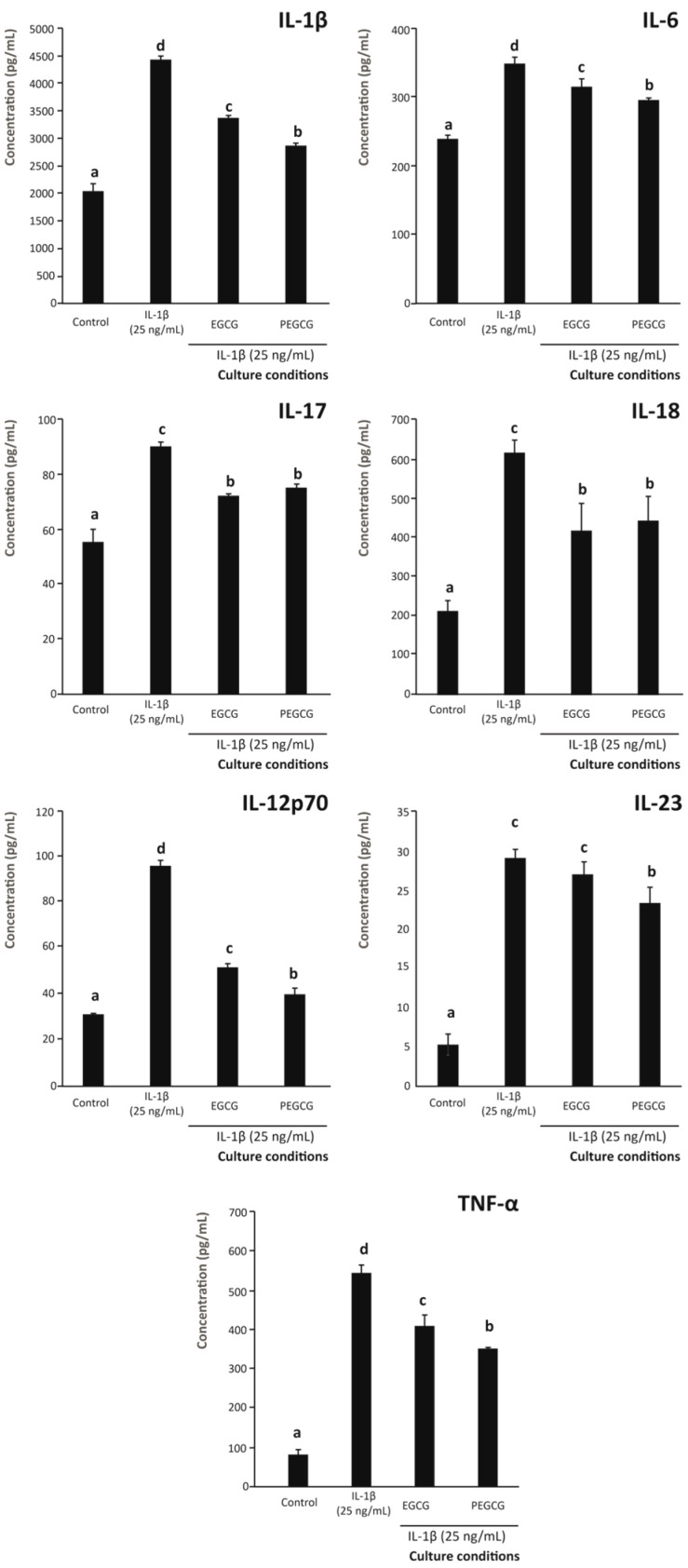
Capacity of the 1 µM EGCG and PEGCG to modulate the secretion of pro-inflammatory interleukins (IL)-1β, IL-6, IL-17, IL-18, IL-12p70, IL-23, and TNF-α by THP-1 monocytic cells differentiated to M0 and exposed to an inflammatory stimulus (25 ng/mL IL-1β). Distinct lowercase letters within each separate bar plot indicate significantly different values at *p* < 0.05 according to one-way analyses of variance (ANOVA) and Duncan’s multiple range test (*n* = 3).

In addition to the modulation of the macrophages’ phenotype, the capacity of EGCG and PEGCG to weaken the differentiation of macrophages towards a phenotype responsible for secreting a range of pro-inflammatory interleukins was determined by monitoring the secretion of IL-1β, IL-6, IL-12p70, IL-17, IL-18, IL-23, and TNF-α (Figure 6), which contribute to the activation of effector cells responsible for tissue damage and chronic inflammation when dysregulated [70]. In this regard, determining M1-related interleukins and their modulation by phytochemicals such as ECGC and its esterification derivatives (PECGC) provided essential insights into the contribution of these bioactive phytochemicals to disease progression or resolution, especially in inflammatory disorders (e.g., IBD). Thus, in this microenvironment, as demonstrated by the evaluation of the macrophage’s capacity to evolve the cell phenotype, the remarkable plasticity of macrophages allowed them to adopt different functional phenotypes, characterised beyond the expression of surface costimulation molecules, by a distinct capacity to secrete pro- and anti-inflammatory interleukins.

When setting up the profile of pro-inflammatory interleukins secreted by M1 macrophages, quantifiable amounts of all of them were recorded (Figure 6), and it was found that their concentration decreased by 26.6%, on average, as a result of Caco-2 pre-treatment with both EGCG and PEGCG, compared to the profile recorded for macrophages exposed to positive control conditions (Figure 6).

However, despite the significant reduction obtained by the treatment of EGCG and PEGCG, no interleukin remained at the baseline values corresponding to the effect of untreated samples (control). As shown in Figure 6, when comparing the efficiency of EGCG and its palmitoyl derivative, no significant difference (*p* > 0.05) was observed for IL-17 and IL-18, for which both bioactive compounds demonstrated matching reducing efficiency compared to the positive control (18.3% and 30.1% higher, respectively). Concerning the expression of the additional interleukins monitored (IL-1β, IL-6, IL-12p70, IL-23, and TNF-α), PEGCG lowered their concentration more efficiently and significantly than EGCG by 14.9%, 6.1%, 22.7%, 13.6%, and 14.0%, respectively (Figure 6).

These results indicated that PEGCG is characterised by a critical capacity to modulate macrophage polarisation and activation relative to its precursor (EGCG), thus contributing more efficiently to the restoration of tissue homeostasis disturbed during inflammation. In this connection, previous research has provided evidence on the effect of EGCG on macrophage polarisation and the pathophysiological consequences in a range of organs using in vitro and in vivo models based on its capacity to decrease the expression of IL-1β, IL-6, and TNF-α in cells exposed to LPS-based inflammatory conditions [71]. Moreover, this flavanol demonstrated the suppression of M1 polarisation in vivo, as determined by the level of IL-1β in mouse serum [72].

However, to date, there is limited evidence on the capacity of EGCG derivatives to alter the secretion of pro-inflammatory interleukins by macrophages. In this regard, the anti-inflammatory activities of EGCG-docosahexaenoic acid (EGCG-DHA) and EGCG-docosapentaenoic acid (EGCG-DPA) have also been reported, using an in vitro inflammatory model that involves LPS-stimulated mouse macrophages. This approach allowed the validation of the attenuation capacity of EGCG-DHA and EGCG-DPA derivatives of inflammatory mediators such as nitric oxide (NO) and prostaglandin E_2_ (PGE_2_) through the suppression of the nuclear factor κB and mitogen-activated protein kinase (NF-κB and MAPK, respectively) pathways, which could imply downstream effects on interleukins via interconnected ones [20,73]. In this context, the present study has demonstrated that PEGCG provides more efficient suppression of the synthesis of IL-1β and IL-12p70, IL-23, and TNF-α by macrophages compared to its unesterified precursor, EGCG. The registration of this enhanced bioactivity represents a significant advancement in the understanding of enhanced immune modulatory activity of (poly)phenol derivatives, in this case, tentatively associated with the amphiphilic character conferred by the fatty acid moiety [14]. Thus, according to these results, PEGCG emerges as a promising candidate for anti-inflammatory therapies in processes mediated by immunological disturbances and autoreactivity, in conditions involving macrophage-driven interleukin release. Based on this evidence, a novel chemical strategy can be outlined to overcome the constraints of EGCG application, associated with its poor stability and bioavailability.

#### 3.3.2. Contribution of PEGCG and EGCG to Macrophage Polarisation Towards Anti-Inflammatory (M2) Cells

**Figure 7:**
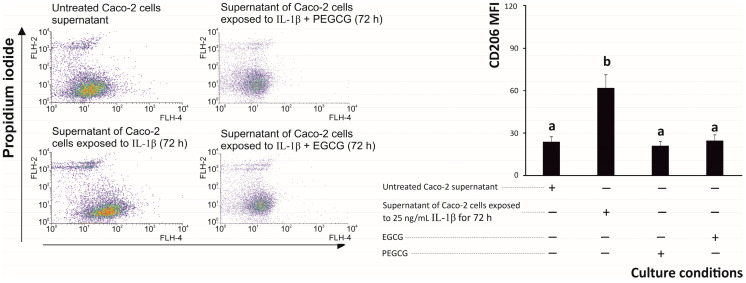
Dot plots of representative anti-inflammatory (CD206-Coralite^®^ Plus 647, M2) macrophages and propidium iodide double stains of M0 macrophages (differentiated from THP-1 monocytic cells treated with Phorbol-Myristate Acetate) exposed to pro-inflammatory growth media of Caco-2 cells in the presence/absence of epigallocatechin gallate and palmitoyl epigallocatechin gallate. Distinct lowercase letters indicate significantly different values at *p* < 0.05 according to one-way analyses of variance (ANOVA) and Duncan’s multiple range test (*n* = 3).

In addition to the inhibition of the macrophagic differentiation to a pro-inflammatory (M1) phenotype, to further understand the enhanced immunomodulatory capacity of PEGCG relative to EGCG, their capacity to induce an anti-inflammatory (M2) phenotype by macrophages under inflammatory growing conditions during 72 h was assessed. The differentiation of M2 cells was monitored by measuring the expression of the cell surface protein CD206 using flow cytometry after exposing THP-1 cells to the interleukin environment generated by intestinal epithelial cells, maintained in inflammatory conditions in the presence/absence of EGCG and its palmitoyl derivative (Figure 7).

The positive control, represented by the interleukin microenvironment generated by epithelial Caco-2 cells under inflammatory conditions, significantly augmented the expression of the M2-marker CD206 in THP-1 differentiated macrophages by almost threefold, which is associated with an anti-inflammatory phenotype [69], after 72 h of exposure compared with untreated cells (Figure 7).

The evolution of the macrophage phenotype observed in the present work during inflammation is in good agreement with previous descriptions of the occurrence of both M1 and M2 types of macrophages during an inflammatory—process, which are activated alternatively to maintain a steady ratio needed for maintaining homeostasis (activation and suppression of the immune response, respectively) in vivo [74]. The supernatant of Caco-2 cells pre-treated with EGCG and PEGCG before exposure to the inflammatory stimulus did not increase the frequency of cells expressing CD206, maintaining the percentage at the level expressed by untreated macrophages (Figure 7). The physiological meaning of both types of macrophages implies that an imbalance between the two types of macrophages could be associated with the development of immune-mediated diseases [74]. In this regard, when analysing the intervention of EGCG and PEGCG in the process of macrophage differentiation during inflammation and the ratio between M1 and M2 cells, it was observed that while macrophages exposed to the growth media of untreated epithelial cells this ratio was 54.7:45.3 (M1/M2), after inflammatory stimulation, the frequency of M1 cells increased to a ratio of 73.5:26.5 (M1/M2). Interestingly, pretreating epithelial cells with EGCG and PEGCG allowed the restoration of the level recorded under control conditions (45.7:54.3 (M1/M2) and 50.9:49.1 (M1/M2), respectively) (Figure 6 and Figure 7). The restoration of the basal distribution between pro- and anti-inflammatory macrophages suggests a valuable capacity of both EGCG and PEGCG bioactive compounds to restore the immune homeostasis [74] and allows them to be highlighted as promising elements of treatments addressed to targeting macrophage polarisation.

**Figure 8:**
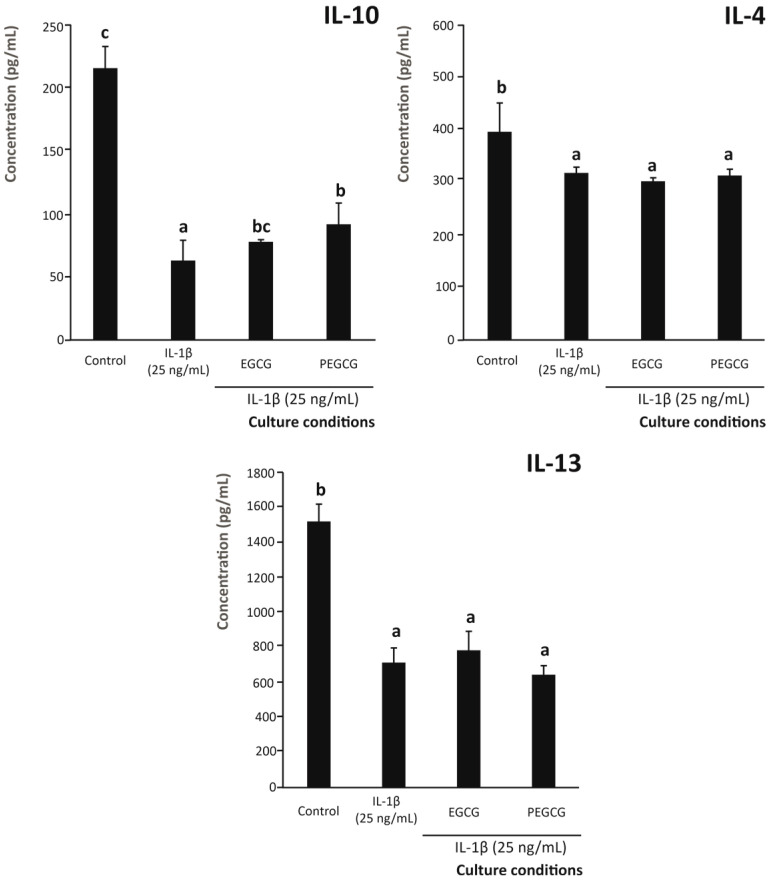
Capacity of the 1 µM EGCG and PEGCG to modulate the secretion of anti-inflammatory interleukins IL-10, IL-4, and IL-13 by THP-1 monocytic cells differentiated to M0 and exposed to an inflammatory stimulus (25 ng/mL IL-1β). Distinct lowercase letters within each separate bar plot indicate significantly different values at *p* < 0.05 according to one-way analyses of variance (ANOVA) and Duncan’s multiple range test (*n* = 3).

Concerning the quantitative profile of interleukins secreted by M2 macrophages, this helps define the differential capacity of EGCG and PEGCG to fine-tune the frequency of anti-inflammatory cells and thus minimise the occurrence of autoimmune diseases. Hence, changes in the secretion of IL-4, IL-10, and IL-13 by M2 macrophages (associated with the promotion of tissue repair and inflammation resolution [74]) by the target phytochemicals (EGCG and its palmitoyl derivative) were monitored (Figure 8). Although the role of interleukins in IBD is not fully understood nowadays because of its complexity and context-dependent nature, the assessed interleukins display important functions to attenuate inflammation, being key regulators that counteract inflammation through distinct but, to some extent, overlapping mechanisms, involving the promotion of M2 macrophage activation, epithelial cell restitution, and mucosal barrier reinforcement [75].

The inflammatory stimulus caused a significant decrease in all anti-inflammatory interleukins (IL-10, IL-4, and IL-13) by 70.6%, 19.7%, and 53.1%, respectively, compared to the control (unstimulated cells) (*p* < 0.05) (Figure 8). Only the expression of the anti-inflammatory IL-10 was significantly (*p* < 0.05) upregulated by EGCG and PEGCG relative to the positive control (34.4%, on average). Despite not matching the level recorded in untreated cells, PEGCG increased the IL-10 concentration to a 17.8% higher extent than EGCG, although this increase did not reach statistical significance (*p* > 0.05) (Figure 8). Conversely, neither EGCG nor PEGCG significantly restored IL-4 and IL-13 levels, at concentrations matching those recorded under inflammatory conditions. These findings are in line with a previous study, which reported that EGCG may play different roles in different environments by promoting the differentiation of macrophages to the M2 phenotype in several organs and tissues in vitro and in vivo [72]. However, no further relevant information is available on the effect of esterifying catechin on the anti-inflammatory interleukin profile.

Concerning the importance of IL-10 in the pathogenesis of IBD, increased levels have been promoted as highly desirable and have been achieved in recent studies [76,77]. The benefits of augmented concentrations of IL-10 are based on the fact that its downregulation enhances the secretion of proinflammatory cytokines by mononuclear phagocytes in vitro [78]. This modified interleukin profile is consistent with the ability of IL-10 to induce an anti-inflammatory macrophage phenotype (beyond the expression of CD206), which involves the metabolic reprogramming of polarised macrophages [79].

## 4. Conclusions

Research on new bioactive compounds to tackle the autoimmune response involved in the IBD pathophysiological process has led to the identification of (poly)phenolic derivatives based on esterification with fatty acids. The modification of this structure, which may be chemical or enzymatic, confers advantages to (poly)phenols identified to date as useful bioactive phytochemicals with anti-inflammatory and immunomodulatory capacities, such as EGCG. This is the case of a palmitoyl monoester derivative. However, the actual advantages of the PEGCG and the immune mechanisms arising from or leading to these diversions are still only partially understood. The present work demonstrates that, despite the lack of a concluding demonstration of the aqueous solubility of the fatty acid derivatives of (poly)phenols that this could constitute a significant constraint for the bioactivity of molecules, the differential capacity of EGCG and PEGCG to fine-tune the pro- and anti-inflammatory interleukin profile secreted by epithelial cells evidences enhanced efficiency concerning the reduction in pro-inflammatory molecules, while appearing as almost negligible when monitoring tolerogenic mediators. Thus, the evidence retrieved suggests the modulation of anti-inflammatory interleukins as a non-central pathway to achieve the enhanced tolerogenic and anti-inflammatory capacity of PEGCG compared with EGCG. Interestingly, as a result of this microenvironment, the treatment of epithelial cells with PEGCG does not significantly modify the migratory response under inflammatory conditions relative to EGCG, but reduces the M1 and M2 phenotypic ratio of migrating cells, reducing the percentage of pro-inflammatory cells more efficiently by the palmitoyl derivative. In summary, these results pave the way not only for a better understanding of the M1–M2 changes in IBD agents but also inform about new phenolic ester derivatives that may be more potent in inhibiting immune-mediated inflammation via M1/M2 modulation, since they identify the advantages of lipophilisation of phenolic compounds to obtain enhanced applications towards functional molecules. Thus, PEGCG emerges as a promising candidate for anti-inflammatory therapies in processes mediated by immunological disturbances and autoreactivity. For this, combining the effects of their polar head with their capability to interfere with membranes could be exploited in several lipophilic bioactive compounds and health-promoting formulations. As a future perspective, the main results described in the manuscript support the relevance of further exploring the molecular mechanisms underlying the observed immunomodulatory activity and elucidating the key molecular interactions responsible for this functionality, whether involving membrane receptors or intracellular targets and conducting dose–response evaluations to retrieve comprehensive evidence on the actual biological scope of EGCG and PEGCG, and the real functional advantages derived from the lipophilisation of phenolics.

## Data Availability

The original contributions presented in this study are included in the article. Further inquiries can be directed to the corresponding author.

## Abbreviations

The following abbreviations are used in this manuscript:

ANOVA	Analysis of variance
BSA	Bovine serum albumin
Caco-2	Human colon adenocarcinoma cell line
COX-2	Cyclooxygenase-2
EDTA	Trypsin-ethylenediaminetetraacetic acid
EGCG	Epigallocatechin gallate
EGCG-DHA	Epigallocatechin gallate-docosahexaenoic acid
EGCG-DPA	Epigallocatechin gallate-docosapentanoic acid
EMEM	Eagle’s minimum essential medium
FBS	Foetal bovine serum
IBD	Intestinal bowel disease
IFN-γ	Interferon gamma
IL	Interleukin
LPS	Lipopolysaccharide
MAPK	Mitogen-activated protein kinase
MIF	Macrophage migration inhibitory factor
NF-κB	Nuclear factor κB
NO	Nitric oxide
PBS	Phosphate-buffered saline
PEGCG	Palmitoyl epigallocatechin gallate
PGE_2_	Prostaglandin E_2_
PMA	Phorbol 12-myristate 13-acetate
RPMI	Roswell Park Memorial Institute Medium
SD	Standard deviation
THP-1	Human monocytic cell line
TNF-α	Tumour necrosis factor-alpha
